# Supplementary material for: IDH3γ functions as a redox switch regulating mitochondrial energy metabolism and contractility in the heart
Source: Nat Commun. 2023 Apr 14;14:2123. doi: 10.1038/s41467-023-37744-x (PMC10102218; doi:10.1038/s41467-023-37744-x)

Supplementary Information
Supplementary tables
Peptide list obtained from the redox proteomics analysis

Peptide containing cysteine	UniProt, accession number	Master protein name	Fold change	p-value
[K].CLDAFPNLR.[D]	P10649	Glutathione S-transferase Mu 1	9.15	0.027
[K].IIFVVGGPGSGKGTQCEK.[I]	Q9R0Y5	Adenylate kinase isoenzyme 1	6.63	0.002
[K].LGRICLDILK.[D]	P61089	Ubiquitin-conjugating enzyme E2 N	5.60	0.000
[R].EKDFIILSCVR.[A]	Q9EPU0	Regulator of nonsense transcripts 1	5.32	0.000
[K].NTEICDELMTR.[H]	Q8VC19	5-aminolevulinate synthase, nonspecific, mitochondrial	4.81	0.008
[R].RVNQAIWLLCTGAR.[E]	P97461	40S ribosomal protein S5	3.98	0.010
[K].EGGPNPENNSSLANILELCR.[S]	Q8K0Z7	Translational activator of cytochrome c oxidase 1	3.33	0.037
[R].VTWDSSFCAVNPR.[F]	Q9WUM4	Coronin-1C	3.32	0.026
[R].VCGAVMETVK.[Q]	Q8VC19	5-aminolevulinate synthase, nonspecific, mitochondrial	3.26	0.050
[K].SLEKVCADLIR.[G]	P60867	40S ribosomal protein S20	3.20	0.038
[K].VTWDSAFCAVNPK.[F]	Q920M5	Coronin-6	3.03	0.026
[R].ITYRELLETTCR.[L]	Q99NB1	Acetyl-coenzyme A synthetase 2-like, mitochondrial	2.80	0.032
[K].VVAACAMPVMKGWNILTNSEK.[S]	Q91VD9	NADH-ubiquinone oxidoreductase 75 kDa subunit, mitochondrial	2.74	0.039
[R].EGVVDIYNCVR.[E]	P28828	Receptor-type tyrosine-protein phosphatase mu	2.73	0.023
[K].VGVGPGSVCTTR.[T]	Q9DCZ1	GMP reductase 1	2.68	0.004
[R].TNGFSLESCR.[S]	O35350	Calpain-1 catalytic subunit	2.65	0.008
[R].VNQAIWLLCTGAR.[E]	P97461	40S ribosomal protein S5	2.62	0.034
[R].VGSVLQEGCEKISQLYGDLKHLK.[T]	Q8K2B3	Succinate dehydrogenase [ubiquinone] flavoprotein subunit, mitochondrial	2.60	0.028
[K].GELLRPSPTCEIK.[A]	A2ASS6	Titin	2.52	0.023
[K].LASLTPGFSGADVANVCNEAALIAAR.[H]	Q8JZQ2	AFG3-like protein 2	2.51	0.029
[K].LTDFGFCAQITPEQSK.[R]	O88643	Serine/threonine-protein kinase PAK 1	2.48	0.033

[R].TTANAIYCPPK.[L]	Q8BG32	26S proteasome non-ATPase regulatory subunit 11	2.43	0.037
[K].ADLSNCLYK.[D]	Q0II04	Nebulette	2.38	0.039
[K].DGVVTIGCIGFPNVGKSSLINGLVGR.[K]	P36916	Guanine nucleotide-binding protein-like 1	2.35	0.029
[K].TTPYQIACGISQGLADNTVVAK.[V]	Q9D0R2	Threonine--tRNA ligase 1, cytoplasmic	2.30	0.033
[R].ASDSSLVCSR.[Y]	Q8BYW9	EGF domain-specific O-linked N-acetylglucosamine transferase	2.24	0.035
[R].VNLVAILCTHK.[H]	Q69ZP3	Probable hydrolase PNKD	2.22	0.005
[R].VAIYMPVSPLAVAAMLACAR.[I]	Q99NB1	Acetyl-coenzyme A synthetase 2-like, mitochondrial	2.18	0.005
[K].ALNVEPDGTGLTCSLAPNILSQL.[-]	P99029	Peroxiredoxin-5, mitochondrial	2.11	0.006
[R].VTNDNTFCR.[L]	P29268	CCN family member 2	2.09	0.046
[K].VPTTLAEYCIK.[T]	Q6ZWZ2	Ubiquitin-conjugating enzyme E2 R2	2.09	0.048
[K].TQAIVCQQLDLTHLK.[E]	P61222	ATP-binding cassette sub-family E member 1	2.08	0.014
[R].TIQFVDWCPTGFK.[V]	Q9JJZ2; P68369; P68373	Tubulin alpha-8 chain	2.08	0.049
[R].LIIGQNGILSTPAVSCIIR.[K]	Q8BZF8	Phosphoglucomutase-like protein 5	2.08	0.039
[K].RPNKPLFTGLVTQCQK.[M]	Q8K4Z3	NAD(P)H-hydrate epimerase	2.07	0.020
[K].DGSASGTTLLEALDCILPPTRPTDKPLR.[L]	P10126	Elongation factor 1-alpha 1	2.04	0.017
[R].GSLLGCSINISDIR.[D]	O35350	Calpain-1 catalytic subunit	2.02	0.009
[K].EDVKSCAEFVSGSQLR.[I]	Q9DCX2	ATP synthase subunit d, mitochondrial	2.02	0.017
[K].KLPVGFTFSFPCR.[Q]	P17710	Hexokinase-1	1.98	0.014
[R].LEALELKECLAHTPK.[L]	O09131	Glutathione S-transferase omega-1	1.97	0.001
[R].SAVQYAECQSK.[A]	Q8K2I4	Beta-mannosidase	1.95	0.036
[K].ELETQLLEQCTVDTGAAK.[G]	Q9QZB7	Actin-related protein 10	1.95	0.050
[R].LDGALCSYTEK.[D]	P52624	Uridine phosphorylase 1	1.94	0.021
[K].FTCEIQGAPNVR.[F]	A2ASS6	Titin	1.94	0.018
[R].ILALCMGNHELYMR.[R]	P26041	Moesin	1.92	0.029
[K].NMMAACDPR.[H]	Q9ERD7; P68372	Tubulin beta-3 chain	1.91	0.002
[R].YVEPIEDVPCGNIVGLVGVDQFLVK.[T]	P58252	Elongation factor 2	1.89	0.045
[R].LVATDGAFSMDGDIAPLQDICR.[L]	O88986	2-amino-3-ketobutyrate coenzyme A ligase, mitochondrial	1.87	0.041
[R].GDSGLYLCK.[A]	A2ASS6	Titin	1.87	0.014
[K].LQAGIDLCETR.[T]	Q8C0M9	Isoaspartyl peptidase/L-asparaginase	1.86	0.049

[R].VFSANSTAACTELAK.[R]	Q9D0M1	Phosphoribosyl pyrophosphate synthase-associated protein 1	1.85	0.002
[R].KNANCSIEESFQR.[F]	P38060	Hydroxymethylglutaryl-CoA lyase, mitochondrial	1.84	0.003
[R].TFVSGACDASIK.[L]	P62880	Guanine nucleotide-binding protein G(I)/G(S)/G(T) subunit beta-2	1.84	0.015
[R].SLVANLAAANCYK.[K]	P55264	Adenosine kinase	1.81	0.016
[K].LVEEAIQCAEK.[I]	Q8BH95	Enoyl-CoA hydratase, mitochondrial	1.80	0.002
[K].VVNEINIEDLCLTK.[A]	Q9CQB5	CDGSH iron-sulfur domain-containing protein 2	1.80	0.029
[R].IVVHMAHALKPGEFGLASICNGGGGASALLIEK.[L]	Q8QZT1	Acetyl-CoA acetyltransferase, mitochondrial	1.79	0.037
[R].GTLLSLSEQELLDCDKVDK.[A]	Q9R013	Cathepsin F	1.78	0.041
[K].AVIFCLSADKK.[C]	Q9R0P5	Destrin	1.76	0.026
[R].VGAFTVVCKDAEEAK.[R]	P05202	Aspartate aminotransferase, mitochondrial	1.75	0.002
[K].IYQLQVLANCR.[A]	P50431	Serine hydroxymethyltransferase, cytosolic	1.74	0.016
[R].NLADCLR.[S]	Q9R1P3	Proteasome subunit beta type-2	1.74	0.041
[K].GSEDLKKHGCTVLTALGTILK.[K]	P04247	Myoglobin	1.73	0.030
[R].AAQLCGAGMAAVVEK.[I]	P17710	Hexokinase-1	1.73	0.046
[K].SSFATPGVNVGLFCSTPAVALGR.[A]	Q9D7J9	Enoyl-CoA hydratase domain-containing protein 3, mitochondrial	1.73	0.013
[R].VVDGIFAVCGVPESK.[L]	Q99KK9	Histidine--tRNA ligase, mitochondrial	1.71	0.012
[R].ILDLTECSAVQFDYSQER.[V]	Q9QXT1	Dual adapter for phosphotyrosine and 3-phosphotyrosine and 3-phosphoinositide	1.71	0.036
[R].MPLGFTFSFPCK.[Q]	P17710	Hexokinase-1	1.70	0.025
[K].TGVGYPQLSAVIECADSAHGLK.[G]	Q9DCZ1	GMP reductase 1	1.70	0.025
[K].LVEEAIQCAEKIASNSK.[I]	Q8BH95	Enoyl-CoA hydratase, mitochondrial	1.69	0.023
[R].DLTTAGAVTQCYR.[D]	P62717	60S ribosomal protein L18a	1.68	0.033
[R].VTNRDIICQIAYAR.[I]	P47962	60S ribosomal protein L5	1.68	0.008
[K].NLDCVVTGLQQGK.[T]	A2ASS6	Titin	1.67	0.002
[K].LPVGFTFSFPCR.[Q]	P17710	Hexokinase-1	1.67	0.036
[R].VGAFTVVCKDAEEAKR.[V]	P05202	Aspartate aminotransferase, mitochondrial	1.67	0.012

[K].ASADLMSYCEEHAR.[S]	Q9DAS9	Guanine nucleotide-binding protein G(I)/G(S)/G(O) subunit gamma-12	1.67	0.009
[R].YFSLGLPTGSTPLGCYK.[K]	Q9CRC9	Glucosamine-6-phosphate isomerase 2	1.66	0.023
[K].AADLVSENCETYEAHMR.[D]	Q9JLI6	Selenocysteine lyase	1.66	0.016
[K].ISHTEEDCLTFK.[V]	P01027	Complement C3	1.66	0.018
[K].IAYPLGLATLGATVCYPAQSVIIAK.[I]	Q78IK4	MICOS complex subunit Mic27	1.65	0.007
[R].AHGSSILACAPLYSWR.[T]	P11688	Integrin alpha-5	1.65	0.001
[K].AVAVEEFCK.[S]	Q6PE15	Palmitoyl-protein thioesterase ABHD10, mitochondrial	1.65	0.001
[K].LAEEESCR.[E]	Q61543	Golgi apparatus protein 1	1.64	0.045
[K].IKNVDCVLLAR.[H]	Q9CQ65	S-methyl-5'-thioadenosine phosphorylase	1.63	0.035
[K].MVAAVACAK.[V]	Q3ULD5	Methylcrotonoyl-CoA carboxylase beta chain, mitochondrial	1.62	0.006
[K].STLIDALCR.[K]	Q64514	Tripeptidyl-peptidase 2	1.62	0.023
[R].VIATFACSGEK.[E]	Q9JLJ2	4-trimethylaminobutyraldehyde dehydrogenase	1.61	0.008
[R].LSMEQICR.[H]	Q6P4S6	Serine/threonine-protein kinase SIK3	1.61	0.021
[R].SLGLICDECPIGYTGPR.[C]	Q60675	Laminin subunit alpha-2	1.59	0.005
[R].LFVSGACDASAK.[L]	P62874	Guanine nucleotide-binding protein G(I)/G(S)/G(T) subunit beta-1	1.59	0.007
[R].EGITFHFTPVLVCK.[D]	Q8VDL4	ADP-dependent glucokinase	1.59	0.039
[R].KGLIAAICAGPTALLAHEVGFGCK.[V]	Q99LX0	Parkinson disease protein 7 homolog	1.59	0.042
[K].LLASCSADMTIK.[L]	P63005	Platelet-activating factor acetylhydrolase IB subunit beta	1.58	0.044
[R].TLSGMESYCVR.[A]	P80315	T-complex protein 1 subunit delta	1.58	0.017
[K].EILGTAQSVGCNVDGR.[H]	P35979	60S ribosomal protein L12	1.56	0.013
[K].LLTQLHYCEK.[A]	Q99KK9	Histidine--tRNA ligase, mitochondrial	1.56	0.027
[K].SAGGIGVAVSCIR.[A]	P07742	Ribonucleoside-diphosphate reductase large subunit	1.56	0.026
[K].VTYCPTEPGTYIINIK.[F]	Q8VHX6	Filamin-C	1.56	0.010
[R].GTFCSFDTPDEAIR.[N]	P61922	4-aminobutyrate aminotransferase, mitochondrial	1.55	0.037
[R].SIQFVDWCPTGFK.[V]	P68368	Tubulin alpha-4A chain	1.55	0.026

[KR].DLVRVCENIPIVLCG NK.[V]	P62827	GTP-binding nuclear protein Ran	1.55	0.043
[K].KGAFDAYVAVGGGSTM	Q8R0N6	Hydroxyacid-oxoacid transhydro mitochondrial	1.55	0.016
[K].FGANAILGVSLAVCKA GAVEK.[G]	P17182	Alpha-enolase	1.55	0.043
[K].IREIADGLCLEVEGK.[M]	P63028	Translationally-controlled tumor protein	1.55	0.019
[R].AALEALGSCLNNK.[Y]	Q9CZN7	Serine hydroxymethyltransferase, mitochondrial	1.54	0.014
[R].ATNLNCSVIADVR.[H]	Q9D1H8	39S ribosomal protein L53, mitochondrial	1.54	0.030
[K].VFCIGPVFR.[A]	Q922B2	Aspartate--tRNA ligase, cytoplasmic	1.54	0.048
[K].GAVHQLCQSLAGK.[N]	Q8BVI4	Dihydropteridine reductase	1.52	0.010
[R].TSLDLYANVIHCK.[S]	P70404	Isocitrate dehydrogenase [NAD] subunit gamma 1, mitochondrial	1.52	0.016
[K].IEEACEIYAR.[A]	Q9DB05	Alpha-soluble NSF attachment protein	1.52	0.031
[R].TLIQNCGASTIR.[L]	P80318	T-complex protein 1 subunit gamma	1.52	0.039
[K].VFADYEEYIKCQDK.[V]	Q9WUB3	Glycogen phosphorylase, muscle form	1.52	0.006
[K].TFESLVDFCK.[T]	Q61425	Hydroxyacyl-coenzyme A dehydrogenase, mitochondrial	1.51	0.010
[K].VIYDKDQFMCGETVP APSTNK.[E]	Q8K0W9	DPH3 homolog	1.50	0.046
[R].LISLNMNFCSR.[E]	Q04519	Sphingomyelin phosphodiesterase	1.50	0.049

Supplementary Table 1: List of cysteine containing peptides that were identified in the redox proteomic screen to show an increased total reversible oxidation demonstrated by a fold change of ≥ 1.5 and a p-value of ≤ 0.05 in the left ventricles of HyPer-DAO mice compared to wild type mice after 7 days of D-ala treatment. P values were calculated by two-tailed unpaired t-test.

Peptide list obtained from the redox proteomics analysis

Peptide containing cysteine	UniProt, accession number	Master protein name	Fold change	p-value
[K].TCVADESAANCDKSLHTLFGDK.[L]	P07724	Albumin	0.50	0.027
[R].MAHAMNEYPDSCAVLVR.[R]	Q9WVQ5	Methylthioribulose-1-phosphate dehydratase	0.49	0.010
[R].EVFGSGTACQVCPVHQILYEGK.[Q]	O35855	Branched-chain-amino-acid aminotransferase, mitochondrial	0.49	0.002
[K].QFSYTHICAGASAFGK.[N]	Q99LC5	Electron transfer flavoprotein subunit alpha, mitochondrial	0.49	0.013
[K].TLTPGGHAEHDGQPYCHKPCYGILFGPK.[G]	Q9DCT8	Cysteine-rich protein 2	0.49	0.003
[K].YGHAACFGLQPGCLR.[Q]	Q8C1A5	Thimet oligopeptidase	0.48	0.003
[R].QCLSFCPAGVPIR.[D]	P0CW02	Lymphocyte antigen 6C1	0.48	0.043
[R].EGICGSCAMNINGGNTLACTR.[R]	Q9CQA3	Succinate dehydrogenase [ubiquinone] iron-sulfur subunit, mitochondrial	0.48	0.006
[R].GHIEDCGHWTQIEKPTEVNQILIK.[W]	P34914	Bifunctional epoxide hydrolase 2	0.47	0.001
[R].VDLEISPDFLAVPVGGHENSHCICGNER.[K]	Q61838	Pregnancy zone protein	0.47	0.019
[R].LNQATVTSNRPGLFYGQCSEICGSNHSFMPIVLEMVPLK.[Y]	P00405	Cytochrome c oxidase subunit 2	0.47	0.016
[R].VLCVALGQLDRPLDLADDGR.[I]	Q6P3E7	Polyamine deacetylase HDAC10	0.47	0.049
[K].TFHETLNCCGSNALTTLTTTILR.[N]	P35762	CD81 antigen	0.46	0.047
[R].GDGQTCYDIDECSEQPSR.[C]	P10493	Nidogen-1	0.46	0.003
[K].QCEAHLGHVFPDGPKPTGQR.[F]	Q78J03	Methionine-R-sulfoxide reductase B2, mitochondrial	0.46	0.031
[K].EKLCYVALDFENEMATAASSSSLEK.[S]	P68033	Actin, alpha cardiac muscle 1	0.46	0.045
[R].ELACDDPEAEQVALLAVDYLNNHLLQGFK.[Q]	P29699	Alpha-2-HS-glycoprotein	0.45	0.048
[R].LDPQLQLHCSDEIANLCAEEAAAQEQTGQVEECLK.[V]	Q61543	Golgi apparatus protein 1	0.45	0.033
[K].MVTMVEIDQMVIDGCK.[K]	P97355	Spermine synthase	0.45	0.036
[R].HYCIVANPVSR.[D]	Q9EQK5	Major vault protein	0.44	0.000
[K].ECCHGDLLECADDRAELAK.[Y]	P07724	Albumin	0.44	0.015
[K].LCMCADGTLFK.[V]	P82349	Beta-sarcoglycan	0.44	0.014

[R].TGQCECQPGITGQHCER.[C]	P02468	Laminin subunit gamma-1	0.44	0.006
[R].FRCPETLFQPSFIGMESAGIHETTYNSIMK.[C]	P68033	Actin, alpha cardiac muscle 1	0.43	0.049
[R].IQPFQNLTLHPACSGLHYSLQLFEGLK.[A]	O35855	Branched-chain-amino-acid aminotransferase, mitochondrial	0.43	0.010
[K].AVLTSQETLFGGSDCTGNFCLFK.[S]	Q921I1	Serotransferrin	0.42	0.050
[R].LAQGICLELSEASSCEEFKK.[E]	Q64314	Hematopoietic progenitor cell antigen CD34	0.42	0.029
[K].SACGNCYLGDAFR.[C]	Q8WTY4	Anamorsin	0.41	0.039
[R].ACLLCSLVK.[T]	P63271	Transcription elongation factor SPT4-A	0.41	0.045
[R].LCGSGFQSIVSGCQEICSK.[D]	Q8BWT1	3-ketoacyl-CoA thiolase, mitochondrial	0.41	0.047
[R].RCESCAPGYEGNPIQPGGK.[C]	Q05793	Basement membrane-specific heparan sulfate proteoglycan core protein	0.40	0.023
[K].SLCPVSWVSAWDDR.[I]	P56391	Cytochrome c oxidase subunit 6B1	0.40	0.017
[R].VGTKCCTLPEDQRLPCVEDYLSAILNR.[V]	P07724	Albumin	0.40	0.007
[R].NECFLQHKDDNPSLPPFERPEAEAMCTSFK.[E]	P07724	Albumin	0.40	0.006
[K].LCYVALDFENEMATAASSSLEK.[S]	P68033	Actin, alpha cardiac muscle 1	0.38	0.027
[R].VALMGGSHGGFLSCHLIGQYPETYSACIAR.[N]	Q8R146	Acylamino-acid-releasing enzyme	0.38	0.045
[K].NCVILPHIGSATYK.[T]	Q91Z53	Glyoxylate reductase/hydroxypyruvate reductase	0.37	0.001
[K].GDCVECMACSDNTVR.[A]	Q924M7	Mannose-6-phosphate isomerase	0.36	0.001
[K].LYDYCDIPLCASASSFECGKPQVEPK.[K]	P20918	Plasminogen	0.36	0.009
[R].SCAPGSDPDSPLCALCVGGNNPAHMCAANNAEGYHGSSGALR.[C]	Q9DBD0	Inhibitor of carbonic anhydrase	0.36	0.047
[K].QDADFPTSLSFQCVNGK.[H]	Q61129	Complement factor I	0.35	0.046
[R].RGTDECAIESIAVAAIPIPK.[L]	P97821	Dipeptidyl peptidase 1	0.35	0.046
[R].KPVVPGHVLVCPLRPVER.[F]	O89106	Bis(5'-adenosyl)-triphosphatase	0.35	0.035
[R].LPCVEDYLSAILNRVCLLHEK.[T]	P07724	Albumin	0.34	0.001
[R].MLHSCTSEGSAYR.[K]	Q9R098	Hepatocyte growth factor activator	0.34	0.028
[R].DFNLDGAPYGYTPFCDSR.[R]	Q6P5E4	UDP-glucose:glycoprotein glucosyltransferase 1	0.33	0.029

[K].TPVSEHVTKCCSGSLVERRPCFSALTVDETYVPK.[E]	P07724	Albumin	0.33	0.028
[K].TCVADESAANCDKSLHTLFGDKLCAIPNLR.[E]	P07724	Albumin	0.33	0.014
[K].VDIQTEDLEDGTCK.[V]	Q80X90	Filamin-B	0.32	0.043
[K].RDCGGAAAVLGAFR.[A]	Q6NSR8	Probable aminopeptidase NPEPL1	0.32	0.030
[R].LTASSTCGLHSPQPYCIVSHLQDEK.[K]	Q61292	Laminin subunit beta-2	0.32	0.005
[R].VTVQAACGNSVLQDSR.[L]	Q9EPR5	VPS10 domain-containing receptor SorCS2	0.32	0.024
[K].LCYHVLGTDQSEDILCAEFPDEPK.[W]	Q9QUR6	Prolyl endopeptidase	0.31	0.012
[R].LCRPCQCNDNIDPNAVGNCNR.[L]	P02468	Laminin subunit gamma-1	0.31	0.044
[R].VSVCAETFNPDEEEEDNDPR.[V]	P12367	cAMP-dependent protein kinase type II-alpha regulatory subunit	0.30	0.006
[K].SDQGEYTCVASSGR.[M]	P35918	Vascular endothelial growth factor receptor 2	0.30	0.018
[K].GEADAMSLDGGFAYVAGHCGLVPVLAENYLSTHSSGR.[L]	Q9DBD0	Inhibitor of carbonic anhydrase	0.30	0.031
[K].TNGDQASCENELLK.[F]	P15208	Insulin receptor	0.30	0.045
[K].IDFDLHDLIPSCER.[T]	Q99P30	Peroxisomal coenzyme A diphosphatase NUDT7	0.30	0.023
[K].DWSFYILAHTEFTPTETDTYACR.[V]	P01887	Beta-2-microglobulin	0.29	0.014
[R].GTVPGSSPCDSSSGTCFCK.[R]	Q61292	Laminin subunit beta-2	0.29	0.049
[R].VFIGKDCIGGCSDLISMQQTGELMTR.[L]	Q9QUH0	Glutaredoxin-1	0.29	0.016
[R].LQIAACSNQDPLQGTTGLIPLLGIDVWEHAYYLQYK.[N]	P09671	Superoxide dismutase [Mn], mitochondrial	0.28	0.019
[R].FQQLVHQMTELCWEK.[C]	Q9WVA2	Mitochondrial import inner membrane translocase subunit Tim8 A	0.28	0.021
[R].DKLCDLLVANNYFTHFFAPK.[N]	Q61702	Inter-alpha-trypsin inhibitor heavy chain H1	0.27	0.024
[R].AVDVLSELSYAPMTPDHFPTLFCK.[E]	Q7TSQ8	Pyruvate dehydrogenase phosphatase regulatory subunit, mitochondrial	0.27	0.008
[R].LCQTCYPLFQQVAIK.[M]	Q8BGT0	Osteopetrosis-associated transmembrane protein 1	0.27	0.037
[R].SICTTVLELLDK.[Y]	P68254	14-3-3 protein theta	0.26	0.027
[K].FQGPTCETCQTCLGVCAEHK.[E]	P09055	Integrin beta-1	0.25	0.032

[R].FRCPSCRHEVVLDR.[H]	Q9ERP3	Tripartite motif-containing protein 54	0.25	0.025
[K].HVTEDCVFIYCQVGDKPYWK.[D]	Q9CQM5	Thioredoxin domain-containing protein 17	0.25	0.016
[R].VGAGGLDGYSVEYCQEGCSEWTPALQGLTER.[R]	O70468	Myosin-binding protein C, cardiac-type	0.25	0.033
[R].QGTHITVVAHSRPVGHCLEAAAVLSK.[E]	Q9D051	Pyruvate dehydrogenase E1 component subunit beta, mitochondrial	0.24	0.010
[K].YHIQVCTTTPCMLRDSDSILETLQR.[K]	Q9D6J6	NADH dehydrogenase [ubiquinone] flavoprotein 2, mitochondrial	0.24	0.009
[R].SQTEEDCTEELFDFLHARDHCVAHKLFK.[N]	P99028	Cytochrome b-c1 complex subunit 6, mitochondrial	0.24	0.027
[K].LCMAALSHQPQEFPTYVEPTNDEICEAFR.[R]	P21614	Vitamin D-binding protein	0.23	0.042
[R].QAVDQISSGFFSPKDPDCFKDVVNMLMYHDR.[F]	Q8CI94	Glycogen phosphorylase, brain form	0.23	0.005
[K].ECCHGDLLECADDR.[A]	P07724	Albumin	0.23	0.013
[K].QYPCTLVGTWNTWYGEQDQAVHLWR.[Y]	O55126	Protein NipSnap homolog 2	0.23	0.022
[K].ISCSFHVTK.[Q]	Q8VE38	Oxidoreductase NAD-binding domain-containing protein 1	0.23	0.019
[R].FSDTCFLDTDGQATCDACAPGYTGR.[R]	Q05793	Basement membrane-specific heparan sulfate proteoglycan core protein	0.22	0.004
[K].TLTPGGHAEHDGKPFCHKPCYATLFGPK.[G]	Q9DCT8	Cysteine-rich protein 2	0.22	0.004
[K].TVQGAFFGVPVYKDHENCISGEDITHNGIVYTPK.[H]	Q61838	Pregnancy zone protein	0.22	0.035
[R].FCLEGMEESGSEGLDELIFAQK.[D]	Q9D1A2	Cytosolic non-specific dipeptidase	0.21	0.044
[K].DLFQCVSFIIPR.[L]	P28665	Murinoglobulin-1	0.20	0.001
[K].LVSEAIAAGIFNDLGSGSNIDLCVISK.[S]	P70195	Proteasome subunit beta type-7	0.20	0.047
[R].EEYQEELEFCEK.[L]	Q99P31	Hsp70-binding protein 1	0.19	0.000
[K].SAGYPGTLIPYRCDLSNEEDILSMFSAVR.[S]	Q3U0B3	Dehydrogenase/reductase SDR family member 11	0.19	0.000
[R].AQCIYMGGSSYTCSCLPGFSGDGR.[A]	P10493	Nidogen-1	0.19	0.000
[K].ILLCTGAIMEEQAAQLLGVK.[M]	Q9CY45	EEF1A lysine methyltransferase 1	0.19	0.000
[R].VELCAQGSPDLAHLDDGPYEAGGEKEQDPR.[L]	Q61738	Integrin alpha-7	0.19	0.000
[R].ACLISMGYDLGEAEFAR.[I]	Q9JI91	Alpha-actinin-2	0.19	0.001
[K].LVQYLRECEDVMDWINDK.[E]	P16546	Spectrin alpha chain, non-erythrocytic 1	0.18	0.000

[R].KICALDDNVCMAFAG LTADAR.[I]	Q9Z2U0	Proteasome subunit alpha type-7	0.18	0.001
[R].QWVLTAAHCFDGIPY PDVWR.[I]	P26262	Plasma kallikrein	0.18	0.000
[K].CGPMVLDALIK.[I]	Q9CQA3	Succinate dehydrogenase [ubiquinone] iron-sulfur subunit, mitochondrial	0.18	0.000
[K].KQEGQYYCTASNR.[A]	Q08481	Platelet endothelial cell adhesion molecule	0.18	0.001
[R].VVQCSDLGLDKVPW DFPPDTTLLDLQNNK.[I]	P28654	Decorin	0.18	0.000
[K].QNCFDDFQCAAEYLIK .[E]	Q9QUR6	Prolyl endopeptidase	0.17	0.003
[R].QCLSSELLDACTFHK.[Y]	Q9DBD0	Inhibitor of carbonic anhydrase	0.17	0.023
[R].IEDDMDGGDWSFCD GR.[L]	Q61739	Integrin alpha-6	0.17	0.001
[R].GVQDIVVGEGTHFLIP WVQKPIIFDCR.[S]	P67778	Prohibitin	0.17	0.049
[R].HYCVILDPMGPDGK.[N]	Q9EQK5	Major vault protein	0.17	0.001
[K].HQHDRVCGDTVFQL QENVK.[D]	Q61738	Integrin alpha-7	0.17	0.001
[R].VAHMEFCYQELCQLA AER.[R]	Q62261	Spectrin beta chain, non-erythrocytic 1	0.17	0.001
[R].VTGPPIFNPVANEVYL NFESSTHCLADR.[Y]	Q07113	Cation-independent mannose-6-phosphate receptor	0.17	0.003
[K].LGCGEGGCGACTVMI SK.[Y]	Q00519	Xanthine dehydrogenase/oxidase	0.17	0.002
[R].QAQLQDAGIYECESK. [T]	P29533	Vascular cell adhesion protein 1	0.17	0.001
[R].TEHSPFHEHVLGALCS LVTDFPQGVR.[E]	Q99P31	Hsp70-binding protein 1	0.17	0.005
[R].DKETPSGFTLDDVIQT GVDNPGHPFIMTVGCVA GDEESYTVFK.[D]	P07310	Creatine kinase M-type	0.17	0.001
[K].DGQIFMSACQDQTIR. [L]	Q91VU6	DDB1- and CUL4-associated factor 11	0.17	0.001
[K].TVCTIAAHEGTLAAITF NSSGSK.[L]	Q8R3E3	WD repeat domain phosphoinositide-interacting protein 1	0.16	0.001
[K].AMLFMCSGSIIHSLAD EQDIR.[K]	P03921	NADH-ubiquinone oxidoreductase chain 5	0.16	0.039
[K].NCTCGLAEELER.[E]	Q8WTY4	Anamorsin	0.16	0.001
[K].LQDPFSVYRCHTIMN CTQTCPK.[G]	Q9CQA3	Succinate dehydrogenase [ubiquinone] iron-sulfur subunit, mitochondrial	0.16	0.004
[R].YNFYLYGCTNQGYQL LR.[S]	P42703	Leukemia inhibitory factor receptor	0.16	0.001
[R].GASIAHCPNSNLSLSS GLLNVLEVLK.[H]	Q9R111	Guanine deaminase	0.16	0.003

[R].LFGDKTCDLLASFK.[D]	Q60854	Serpin B6	0.16	0.007
[K].KPGVFIPSCDEDGYYR.[K]	Q9ER58	Testican-2	0.16	0.004
[K].FAPVCSICENPIIPR.[D]	Q71FD7	Filamin-binding LIM protein 1	0.16	0.004
[K].NILSHDPDVGTDTMEKEEKTHHACQEMELK.[V]	Q68FE8	Zinc finger protein 280D	0.16	0.001
[K].ASAFNSWFENAEEDLTDPVRCNSLEEIK.[A]	P16546	Spectrin alpha chain, non-erythrocytic 1	0.16	0.002
[K].AAFDIFVLGAEDGCISTKELGK.[V]	P19123	Troponin C, slow skeletal and cardiac muscles	0.16	0.003
[K].LCYGLNMDFVDPAQITMK.[V]	P07742	Ribonucleoside-diphosphate reductase large subunit	0.16	0.004
[K].ALGFPESLVIQAYFACEK.[N]	P54726	UV excision repair protein RAD23 homolog A	0.16	0.003
[K].RLDLTYSFLGSQGVGQCYDSSPCER.[Q]	Q05793	Basement membrane-specific heparan sulfate proteoglycan core protein	0.16	0.004
[K].QCIVAHPVNPPYYVPLVELVPHPETAPATMDR.[T]	Q99KP3	Lambda-crystallin homolog	0.15	0.002
[R].VFIWTCDDASGNMWSPK.[L]	Q9D1M0	Protein SEC13 homolog	0.15	0.002
[K].GASSSSCSETYCGLYPESEPEVK.[A]	Q9JHH6	Carboxypeptidase B2	0.15	0.002
[K].LGRPSLSSEVGVIICDISNPASLDEMAK.[Q]	Q8R127	Saccharopine dehydrogenase-like oxidoreductase	0.15	0.019
[R].VAGEPAADGTVACPCCQAPTRPQALSTNLQLSR.[L]	Q1XH17	Tripartite motif-containing protein 72	0.15	0.002
[R].SSNLLGECSPAQR.[E]	Q9QY81	Nuclear pore membrane glycoprotein 210	0.15	0.018
[R].NLHMEQAPVDLDQPTSSFHVGTCFANAESGTYFDGTGFAK.[A]	Q60675	Laminin subunit alpha-2	0.15	0.002
[R].QLEPGLQGILITCNMNER.[K]	Q99J36	THUMP domain-containing protein 1	0.15	0.015
[K].TCEWIHDSSLSASCK.[E]	Q61207	Prosaposin	0.15	0.006
[K].FFGEFTGFVDMCVQHIPSPK.[V]	O08810	116 kDa U5 small nuclear ribonucleoprotein component	0.15	0.003
[R].WCQSMQDPSASLLER.[Q]	Q9DC50	Peroxisomal carnitine O-octanoyltransferase	0.15	0.003
[K].GVCTEAGMYALR.[E]	P62196	26S proteasome regulatory subunit 8	0.14	0.022
[K].GSFPLDHFGECK.[S]	Q8K0C8	Cytochrome c oxidase assembly protein COX19	0.14	0.019
[R].AFWRELVECFQKISK.[D]	O35459	Delta(3,5)-Delta(2,4)-dienoyl-CoA isomerase, mitochondrial	0.14	0.005
[R].LDSHSCLEVTAATLR.[R]	Q8VCT3	Aminopeptidase B	0.14	0.003

[K].TCNCETEDYGEK.[F]	Q8VEK3	Heterogeneous nuclear ribonucleoprotein U	0.14	0.011
[K].TYAACPQNWIGVENK.[C]	Q91V08	C-type lectin domain family 2 member D	0.14	0.012
[R].EAGTWMDDTCDSK.[Q]	Q61830	Macrophage mannose receptor 1	0.14	0.006
[K].DMFNVNVLALSICTR.[E]	Q3U0B3	Dehydrogenase/reductase SDR family member 11	0.14	0.016
[R].LPLTLAGASYEGVAVNDCIESGR.[Q]	P51175	Protoporphyrinogen oxidase	0.14	0.010
[K].VCQCDSNGDCTDVDR.[I]	P15116	Cadherin-2	0.14	0.034
[R].TKCDEWSIISEGKIECESAETTEDCIEK.[I]	Q921I1	Serotransferrin	0.14	0.043
[R].ECSLQVAEDELVSTLK.[H]	P21126	Ubiquitin-like protein 4A	0.13	0.011
[K].FCASGPYGGEDCPQWMVPITISTSEDPNQAK.[L]	Q11011	Puromycin-sensitive aminopeptidase	0.13	0.020
[R].IMRPTDVPDQGLLCDLLWSDPDKDVQGWGENDR.[G]	P62137	Serine/threonine-protein phosphatase PP1-alpha catalytic subunit	0.13	0.001
[R].DSSTCPGDYVLSVSENSR.[V]	P47941	Crk-like protein	0.13	0.006
[K].DCSIYGEDTVTDEEGK.[F]	Q6GQT9	Nodal modulator 1	0.13	0.013
[R].GADCCVLVFDVTAPNTFK.[T]	P51150	Ras-related protein Rab-7a	0.13	0.007
[R].VGQILHPEECMYAVGQGALAVEVR.[A]	P22907	Porphobilinogen deaminase	0.13	0.013
[K].GEDFYCVTCHETK.[F]	P97447	Four and a half LIM domains protein 1	0.13	0.008
[R].FIAHGDADVMVAGGTDSCISPLSLAGFSR.[A]	Q9D404	3-oxoacyl-[acyl-carrier-protein] synthase, mitochondrial	0.13	0.041
[K].LVDGCYSFWQAGLLPLLHR.[A]	Q8K2I1	Protein farnesyltransferase subunit beta	0.13	0.009
[K].LQSLDLSAVAQMTCGTPPGADCSESECGGPNCR.[T]	P02469	Laminin subunit beta-1	0.12	0.020
[K].DVDYVCISDNYWLGK.[N]	Q9D1A2	Cytosolic non-specific dipeptidase	0.12	0.022
[R].VCMVYDLYPTLTPLAVAYAR.[A]	Q9CWJ9	Bifunctional purine biosynthesis protein ATIC	0.12	0.008
[R].LSVSPLPTLTEDDELLCLFGDSPPHPAR.[V]	B2RXS4	Plexin-B2	0.12	0.011
[R].EGQDATACEVNPLACLSQTATCAPEIPAFSHGGFAYR.[D]	O70423	Membrane primary amine oxidase	0.12	0.032
[K].ECCHGDLLECADDRAELAKYMCENQATISSK.[L]	P07724	Albumin	0.12	0.023

[R].FSFCFSPEPEAEAQAAAGPGPCER.[L]	Q64337	Sequestosome-1	0.12	0.024
[K].FPVFNMSYNPAENAVLLCTR.[A]	Q8CIE6	Coatomer subunit alpha	0.12	0.028
[K].QFLECAQNQSDVKLCEGFNEVLR.[Q]	Q9D1L0	Coiled-coil-helix-coiled-coil-helix domain-containing protein 2	0.11	0.005
[R].IIPLEQCQSYFDMK.[T]	Q9Z319	Atrial natriuretic peptide-converting enzyme	0.11	0.029
[K].DFAPGKPLKCVIKHPNGTQETILLNHTFNETQIEWFR.[A]	Q99KI0	Aconitate hydratase, mitochondrial	0.11	0.017
[K].ILVEGIGEVQEYVDVCDYAAGLSR.[M]	Q9DBF1	Alpha-aminoadipic semialdehyde dehydrogenase	0.11	0.033
[R].DDILCPDCGKDI.[-]	O70433	Four and a half LIM domains protein 2	0.10	0.007
[K].TLTSGGHAEHEGKPYCNHPCYSAMFGPK.[G]	P63254	Cysteine-rich protein 1	0.10	0.030
[K].LCLSEIHK.[M]	P15208	Insulin receptor	0.10	0.031
[R].WLLLCNPGLAEIIVER.[I]	Q8CI94	Glycogen phosphorylase, brain form	0.09	0.040
[K].FVTAVATGGPDNQVHFEGYQVSNQCMALVR.[D]	P60670	Nuclear protein localization protein 4 homolog	0.09	0.048
[K].GCEECFCSGVSNR.[C]	Q60675	Laminin subunit alpha-2	0.09	0.000
[-].MEESEYESVLCVKPEVHVYR.[I]	Q9D1J1	Adaptin ear-binding coat-associated protein 2	0.09	0.033
[R].NSDVMDCVILDDGGFLLMANHDDYTNQIGR.[F]	O08532	Voltage-dependent calcium channel subunit alpha-2/delta-1	0.09	0.040
[K].SHCAEPFTEYWTCLDYSNMQLFR.[H]	Q9DCJ5	NADH dehydrogenase [ubiquinone] 1 alpha subcomplex subunit 8	0.08	0.009
[R].KNGLMDHEDFRACLISMGYDLGEAEFAR.[I]	Q9JI91	Alpha-actinin-2	0.08	0.040
[R].SQSVRPGADVTFICTAK.[S]	Q05793	Basement membrane-specific heparan sulfate proteoglycan core protein	0.07	0.039
[K].SEPDKADPFSFEGPEIVDCDGCTIDWK.[K]	Q78ZA7	Nucleosome assembly protein 1-like 4	0.07	0.040
[R].VVYLQYPSLAPHHQCSQLFLYDWYTK.[V]	Q8R146	Acylamino-acid-releasing enzyme	0.07	0.008
[K].TCGFDFSGALEDISKIPEQSVLLLHACAHNPTGVDPRPEQWK.[E]	P05202	Aspartate aminotransferase, mitochondrial	0.06	0.009
[K].LFQEEFPGIPYPPDAAVECHRGECQSEGVLFFQGNR.[K]	Q91X72	Hemopexin	0.06	0.003

Supplementary Table 2: List of cysteine containing peptides that were identified in the redox proteomic screen to show a decreased total reversible oxidation demonstrated by a fold change of ≤

0.5 and a p-value of ≤ 0.05 in the left ventricles of HyPer-DAO mice compared to wild type mice after 7 days of D-ala treatment. P values were calculated by two-tailed unpaired t-test.

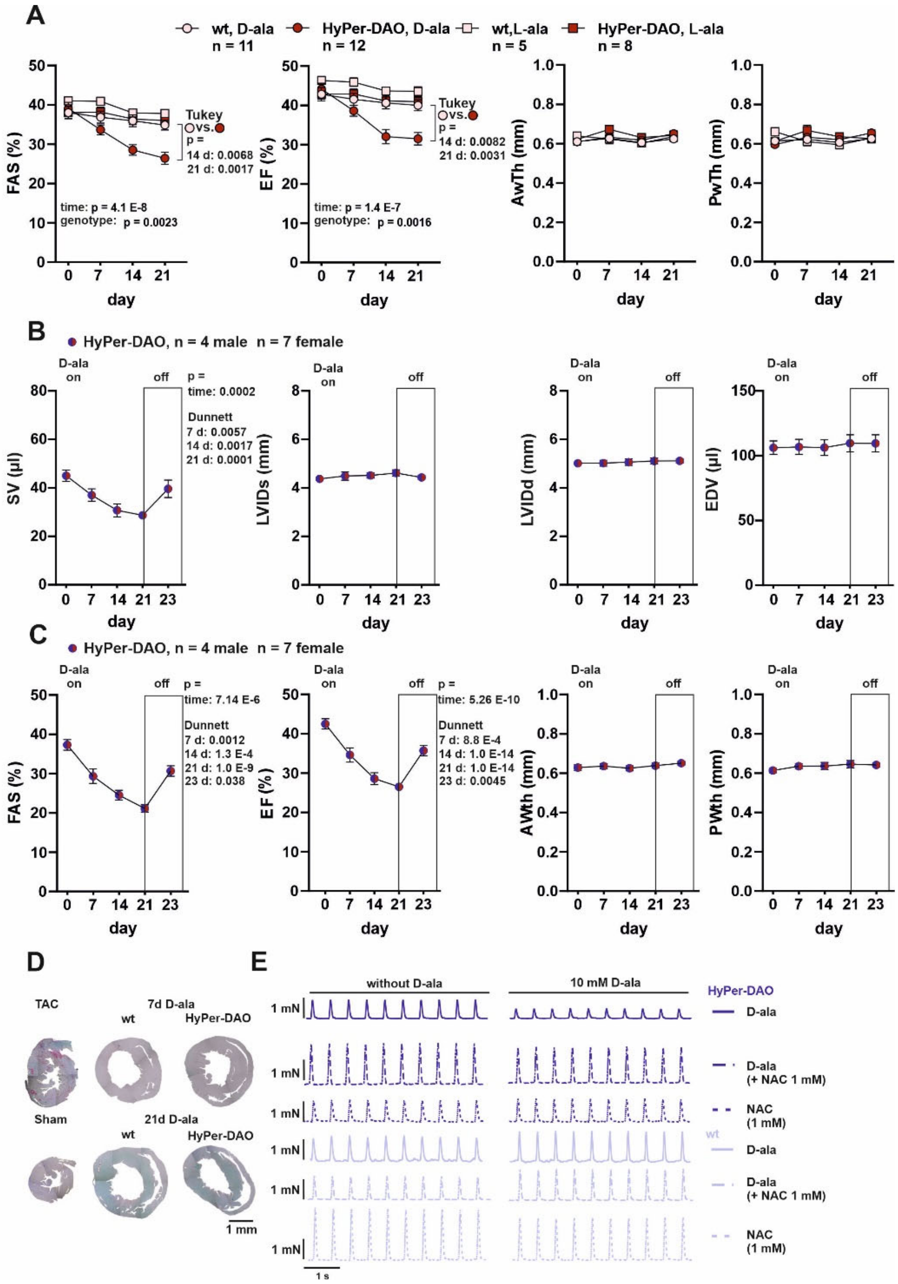

Supplementary Figure 1: Echocardiographic analysis and cardiac fibrosis in HyPer-DAO mice, related to Figure 2 (A, B and C) Echocardiographic analysis of fractional area shortening (FAS), ejection fraction (EF), anterior wall thickness (AWth) and posterior wall thickness (PWth), stroke volume (SV), left ventricular inner diameter in systole (LVIDs), left ventricular inner diameter in diastole (LVIDd), and enddiastolic volume (EDV) in female (in A) and a mixed group of female and male (in B and C) HyPer-DAO or wild type (wt) mice after treatment with D-ala or L-ala in the drinking water as indicated. **(D)** Representative figures of Sirius red/Fast green stained cardiac slices of HyPer-DAO mice after 7 or 21 days of D-ala treatment and mice after TAC or sham surgery. For each mouse the staining was performed once. **(E)** Original tracings of force development measured in heart slices from HyPer-DAO and wt mice after treatment 10 mM D-ala +/- 1 mM NAC. mean ± SEM, one-way ANOVA (B and C) or two-way ANOVA (A). Source data are provided as a Source Data file.

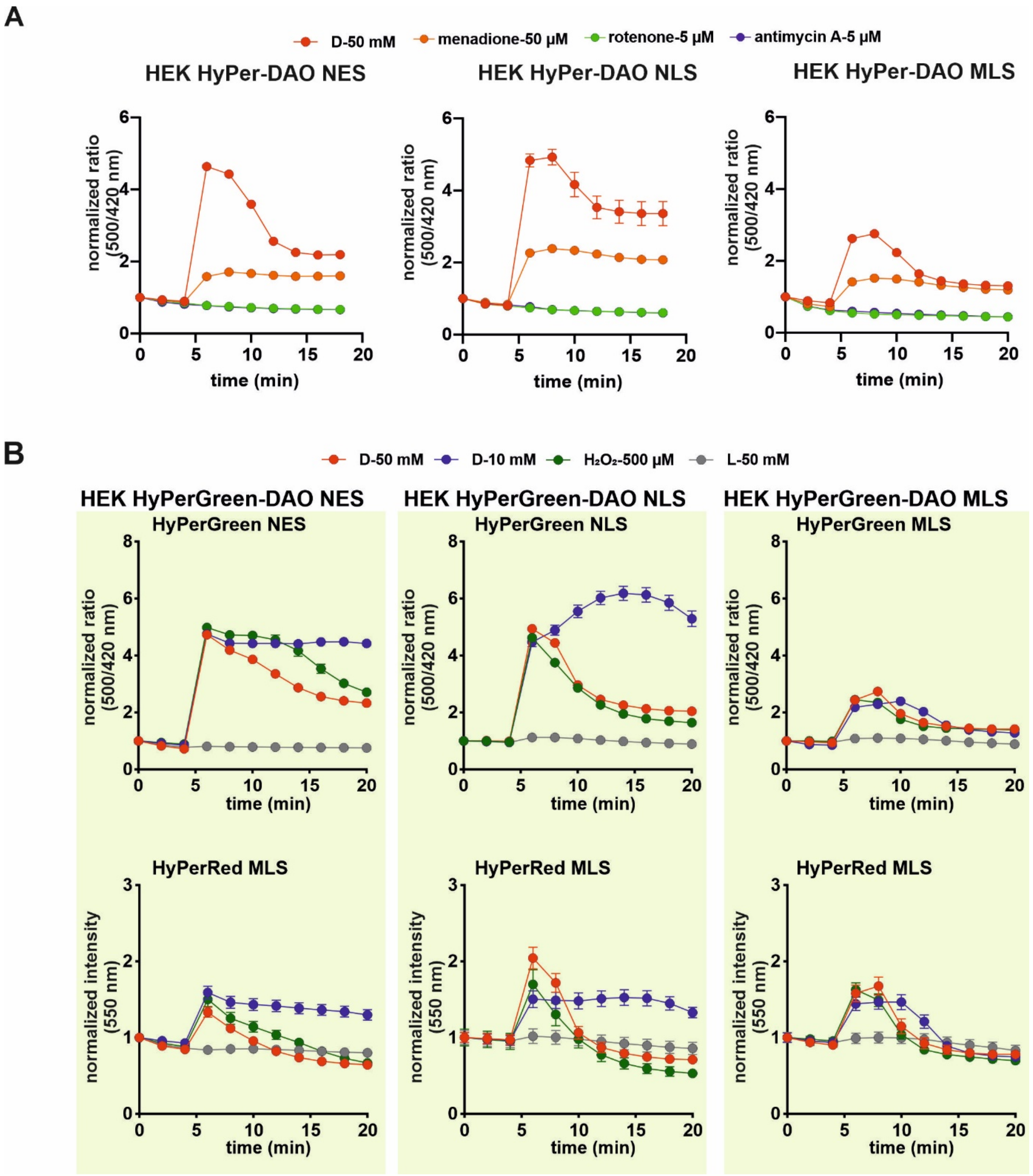

Supplementary Figure 2: Intracellular distribution of H_2O_2 in HEK cells, related to Figure 4A-C

(A) HyPer-Green fluorescence responses were recorded after treating HyPer-DAO-NLS cells with D-ala (n = 42 independent cells), menadione (n = 44 independent cells), rotenone (n = 42 independent cells) or antimycin A (n = 40 independent cells), which were added at time point 4 min. **(B)** HEK cells overexpressing HyPer-DAO fusion protein in the cytoplasm (HyPer-DAO-NES), the nucleus (HyPer-DAO-NLS) and the mitochondrial matrix (HyPer-DAO-MLS) were transiently transfected to overexpress the H_2O_2 sensor HyPerRed localized to the mitochondrial matrix (HPerRed-MLS). The HyPer Green and HyPer Red fluorescence responses were recorded after stimulation with D-ala, L-ala or H_2O_2, which were added at time point 4 min. Ratios are normalized to the HyPer ratio prior treatment, n = 25 independent cells per condition were analyzed. Data are presented as mean values ± SEM. Source data are provided as a Source Data file.

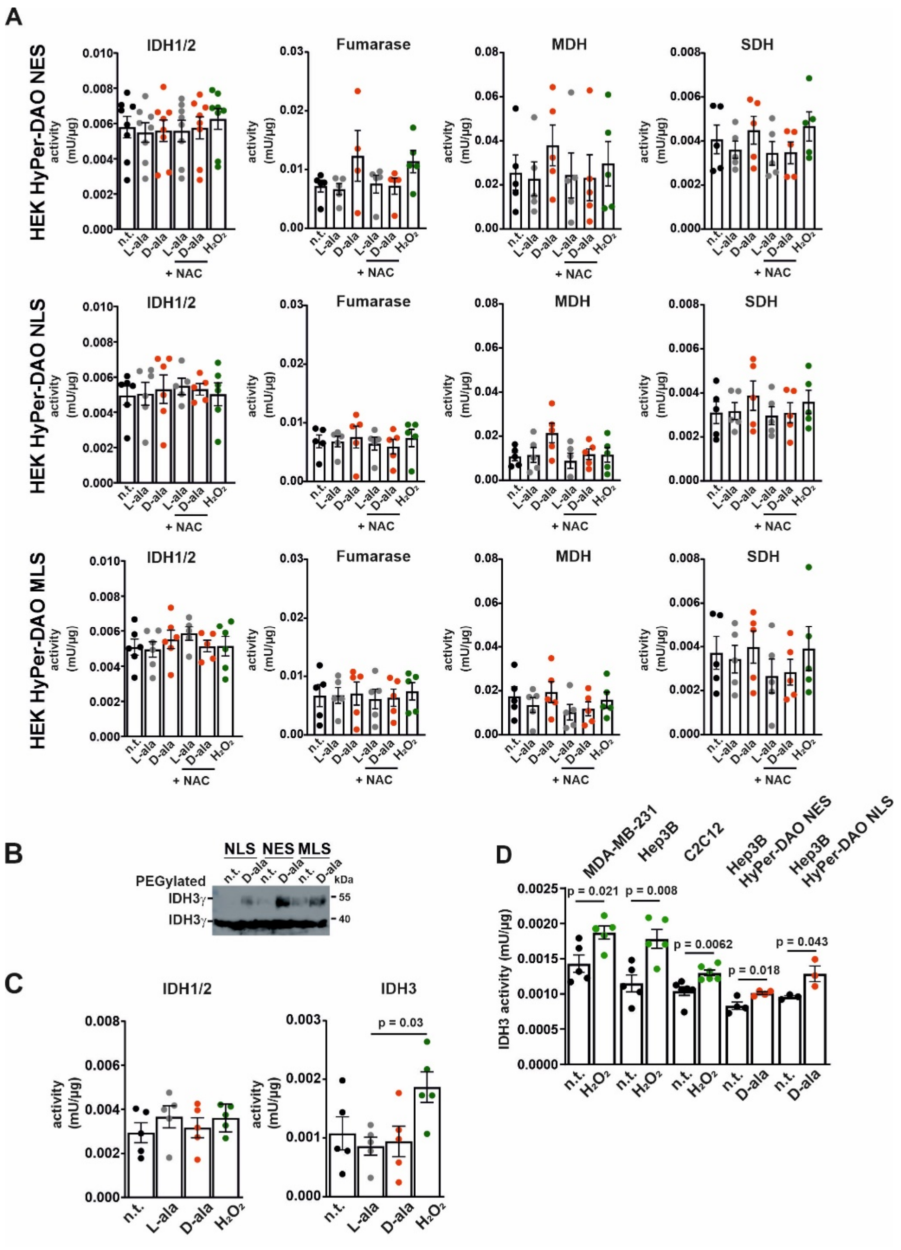

Supplementary Figure 3: TCA cycle enzyme activities in HyPer-DAO overexpressing HEK cells, related to Figure 4 D-G

(A) IDH1/2, fumarase, malate dehydrogenase (MDH) and succinate dehydrogenase (SDH) activities in cell extracts obtained from HEK HyPer-DAO-NES, HEK HyPer-DAO-NLS and HEK HyPer-DAO-MLS cells either non-treated (n.t.) or after treatment with 50 mM D-ala, 50 mM L-ala ± 8 mM NAC or 500 µM H_2O_2 for 20 min. IDH1/2 activity: n = 8 independent experiments for HyPer-DAO NES, n = 6 for HyPer-DAO NLS and MLS except for n = 5 for D-ala and L-ala + NAC, n = 5 independent experiments for all other conditions. **(B)** Representative immunoblot for IDH3γ oxidation analyzed by PEG switch assay in HEK HyPer-DAO-NLS, HEK HyPer-DAO-NES and HyPer-DAO-MLS cells n.t. and after treatment with 50 mM D-ala for 20 min. The experiment has been performed three times. **(C)** IDH1/2 and IDH3 activity in cell extracts obtained from HEK wt cells either non-treated (n.t.) or after treatment with 50 mM D-ala, 50 mM L-ala or 500 µM H_2O_2 µM for 20 min, n = 5 independent experiments. **(D)** IDH3 activity in cell extracts obtained from MDA-MB-231, Hep3B, C2C12, Hep3B HyPer-DAO-NES and Hep3B HyPer-DAO-NLS cells either non-treated (n.t.) or 20 min treatment with 500 µM H_2O_2 for MDA-MB-231, 250 µM H_2O_2 for Hep3B, 5 µM H_2O_2 for C2C12 and 5 mM D-ala for Hep3B HyPer-DAO-NES and -NLS, respectively. 5, 5, 6, 4 and 3 independent experiments for MDA-MB-231, Hep3B, C2C12, Hep3B HyPer-DAO-NES and HyPer-DAO-NLS cells, respectively. Data are presented as mean values ± SEM, one-way ANOVA (A and C) or two tailed unpaired t-test (D). Source data are provided as a Source Data file.

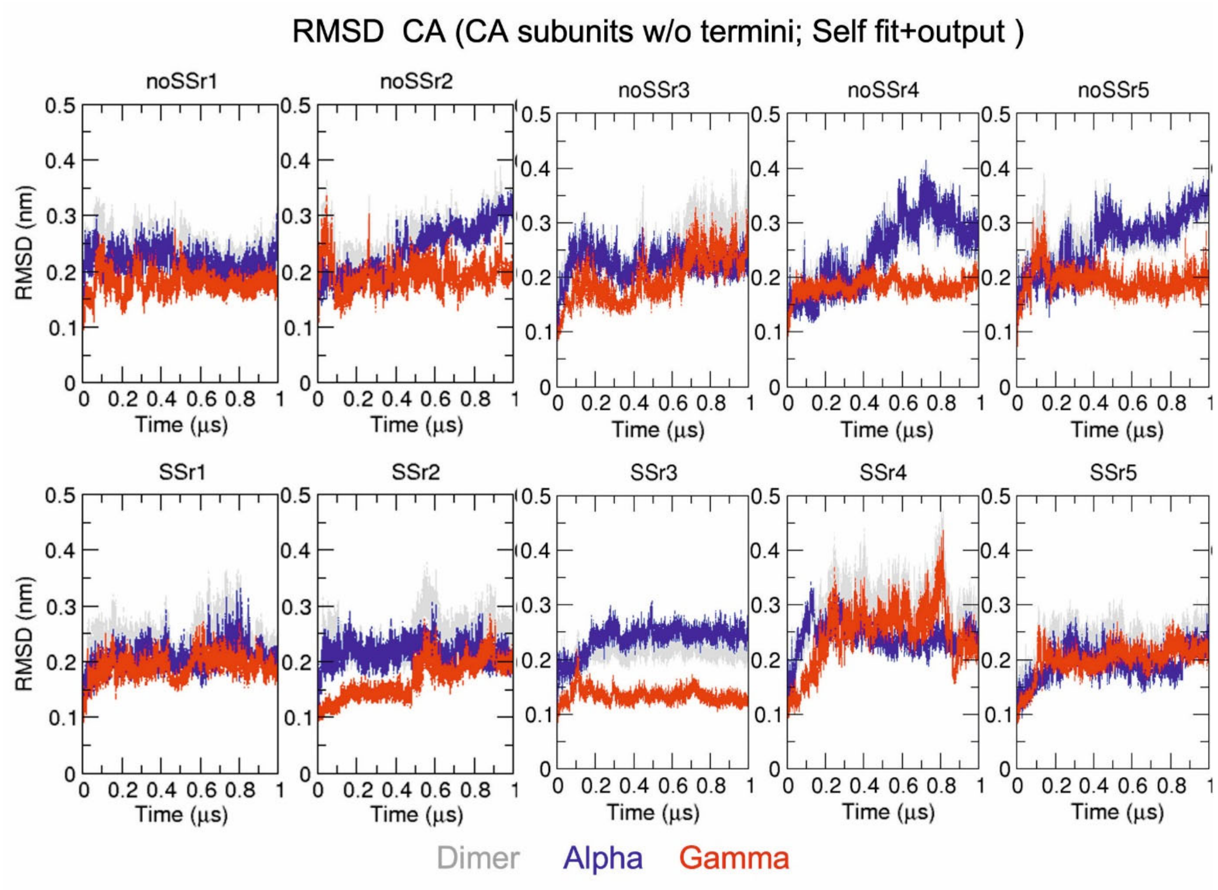

Supplementary Figure 4: Root mean square deviation (RMSD) computed over the Cα-atoms for all MD simulations, related to Figure 6

Curves show results separately for the whole dimer (without disordered termini, grey), the α subunit (blue), and the γ subunit (red).

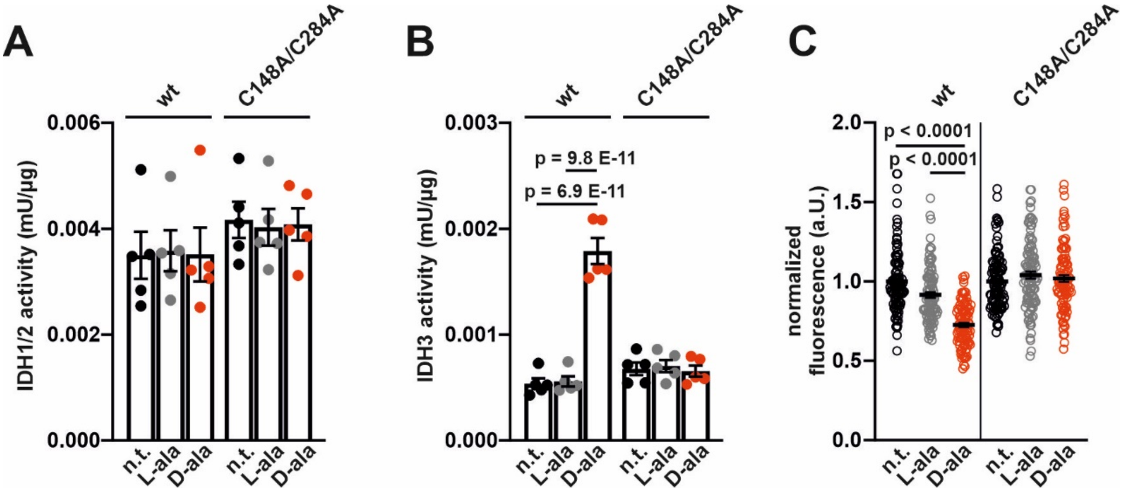

Supplementary Figure 5: Point mutation of IDH3γ Cys148 and Cys284 prevents H_2O_2 induced IDH3 activity and impaired ATP production in the mitochondria, related to Figure 7.

IDH1/2 **(A)** and IDH3 **(B)** activity in cell extracts obtained from HEK HyPer-DAO-NLS wild type (wt) or C148A/C284A cells non-treated (n.t.) and after treatment with 50 mM D-ala or L-ala for 20 min, n = 5 independent experiments per condition. **(C)** Mitochondrial ATP levels determined with the fluorescence sensor ATP-red in HEK HyPer-DAO NLS wt and C148A/284A cells n.t. and after treatment with 50 mM D-ala or L-ala for 20 min. n = 100 cells per condition were analyzed. Data are presented as mean values ± SEM, one-way ANOVA (B and C). Source data are provided as a Source Data file.

Non-cropped blots

Figure 1B

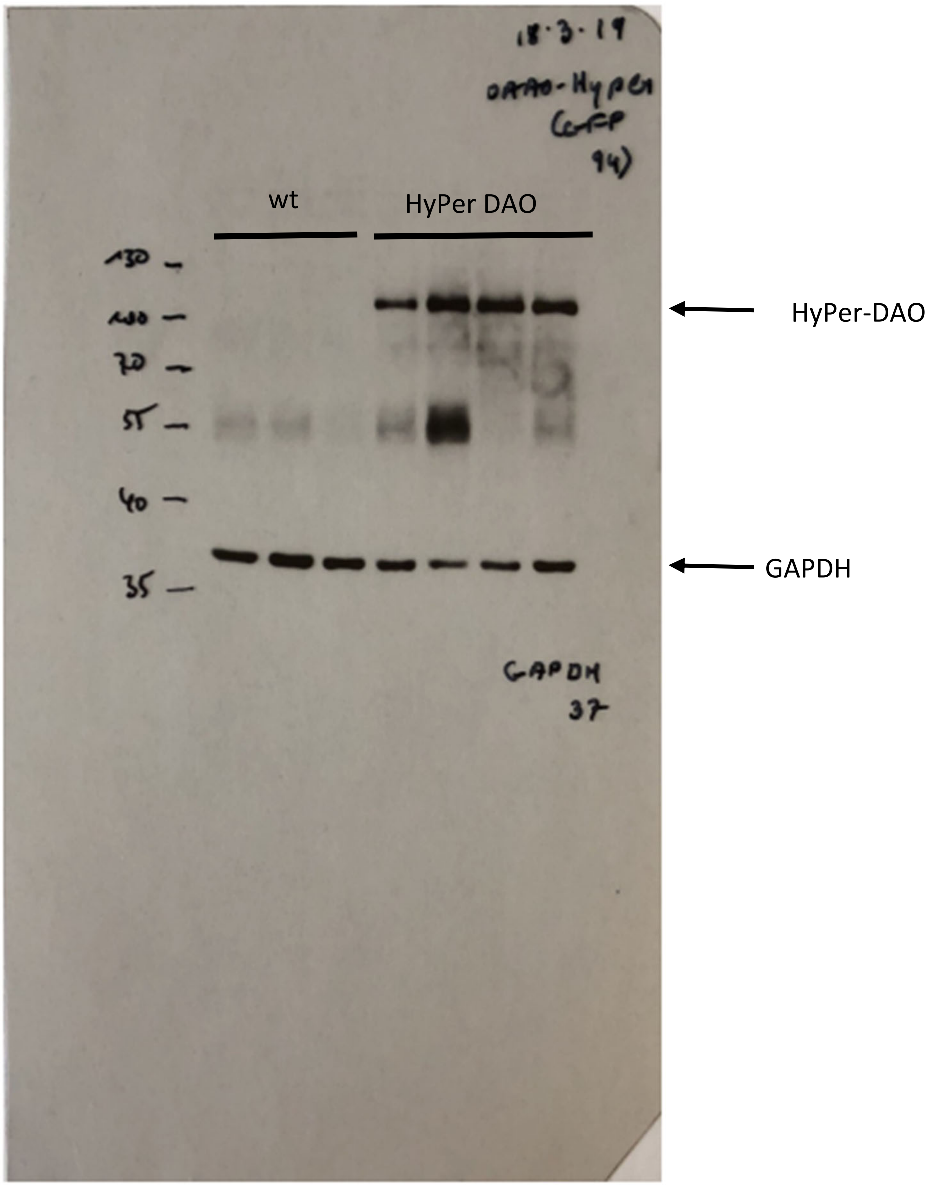

Figure 1C

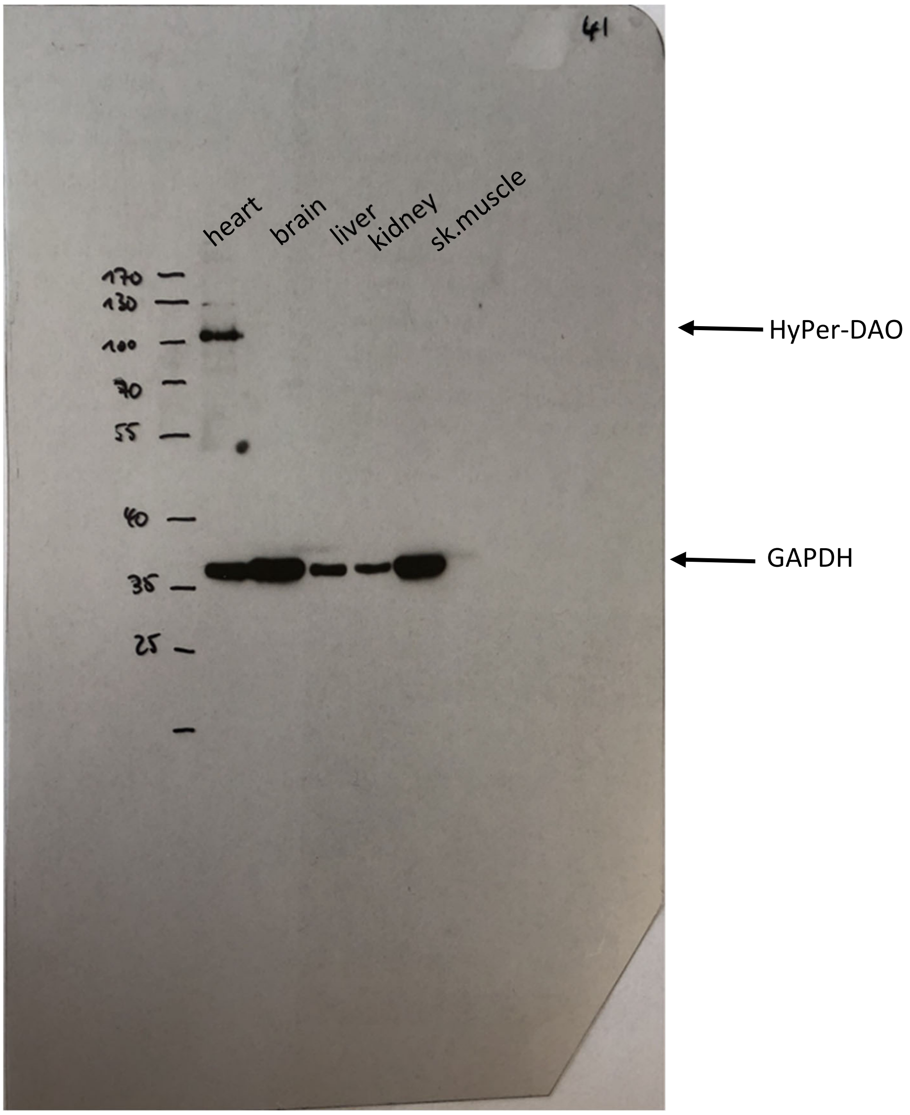
41
heart
brain
liver
kidney
sk.muscle
170
130
100
70
55
40
35
25
HyPer-DAO
GAPDH

Figure 3G

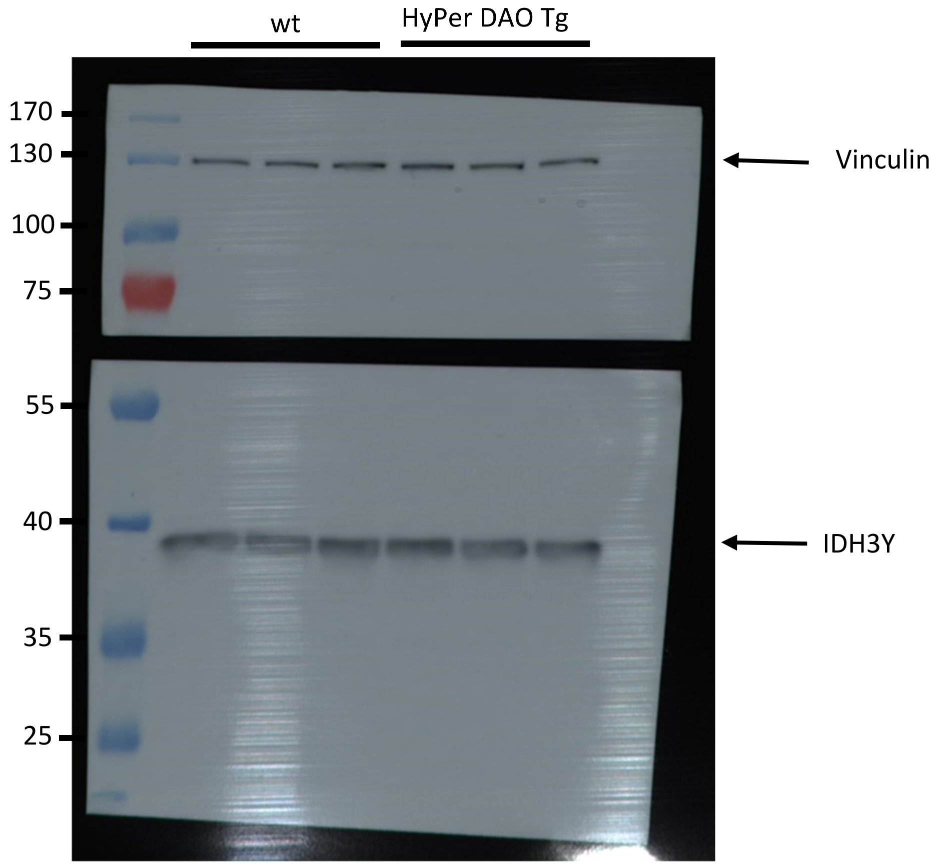

Figure 3K

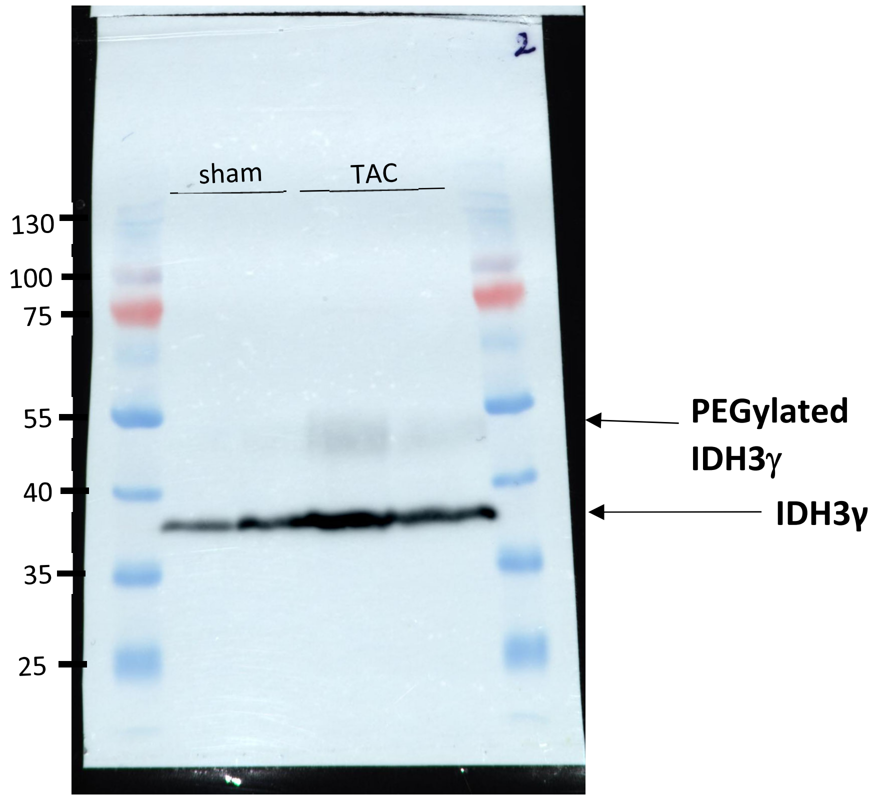

Figure 4E

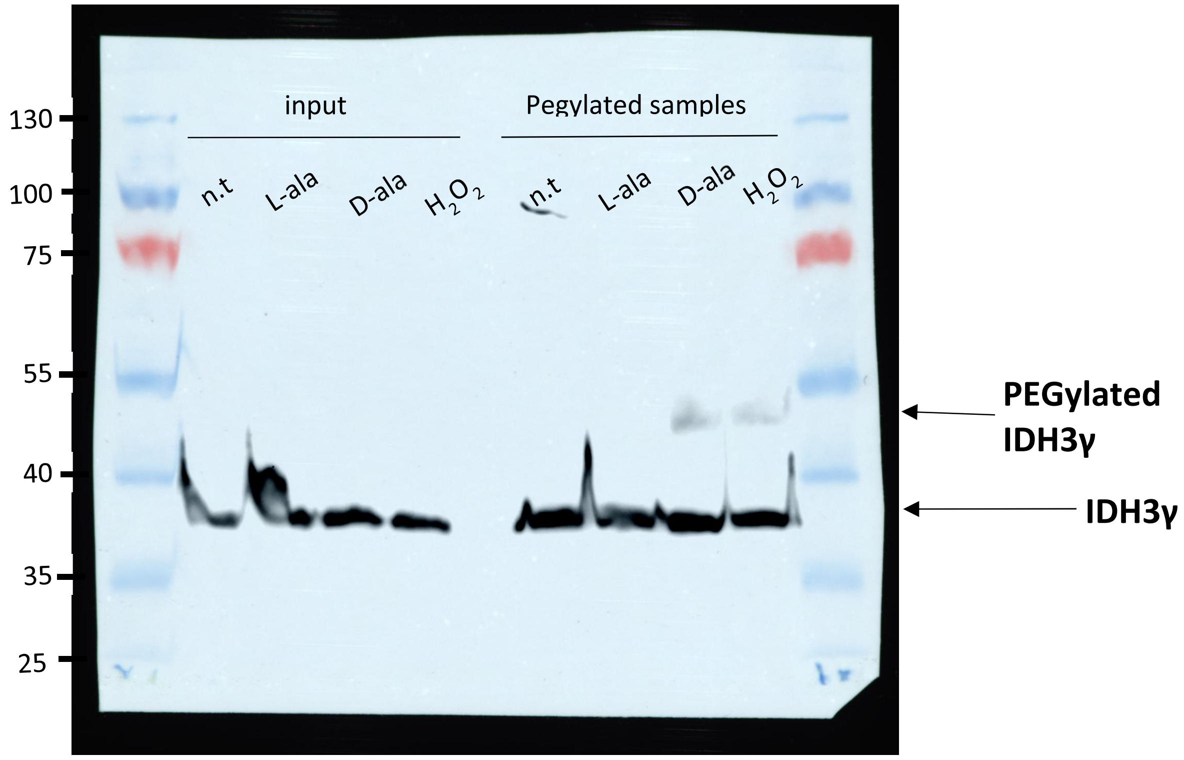

130
100
75
55
40
35
25
input
Pegylated samples
n.t
L-ala
D-ala
H_2O_2
n.t
L-ala
D-ala
H_2O_2
PEGylated IDH3γ
IDH3γ

Figure 4G

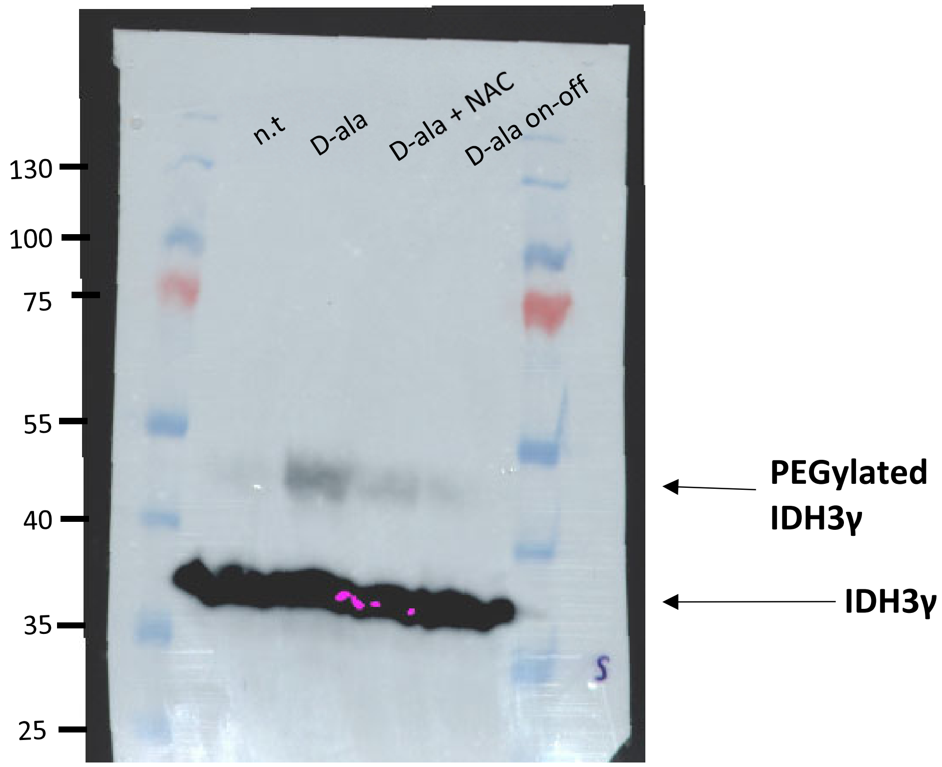

n.t
D-ala
D-ala + NAC
D-ala on-off
130
100
75
55
40
35
25
PEGylated IDH3γ
IDH3γ

Figure 7C

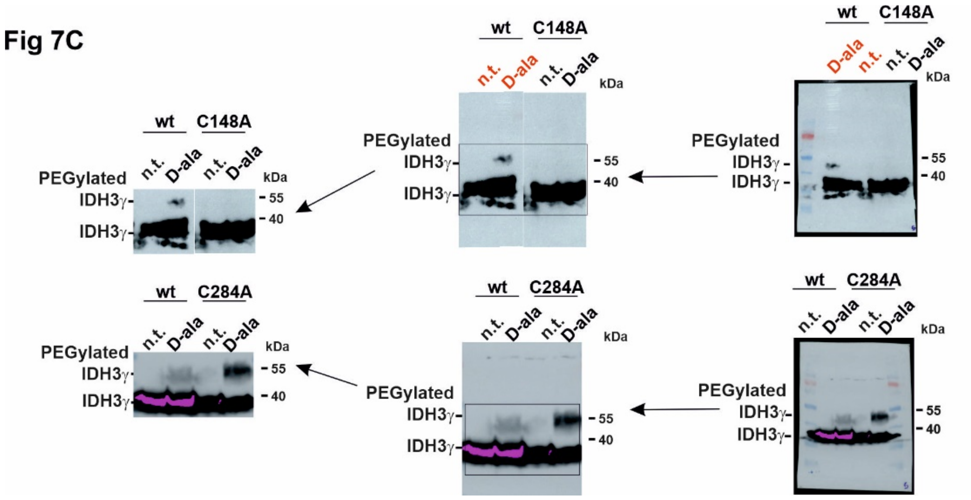

Fig 7C
wt
C148A
n.t.
D-ala
kDa
PEGylated
IDH3γ
IDH3γ
55
40
wt
C284A

Supplementary Figure 3B

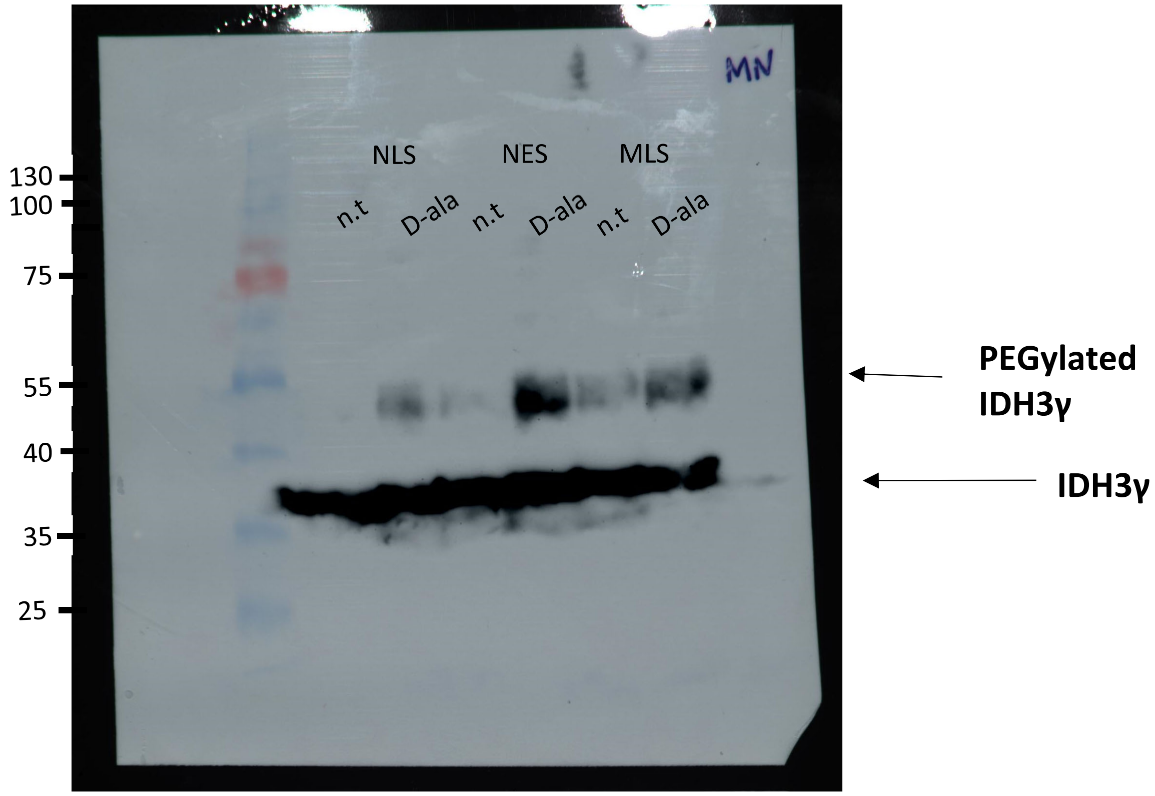

Supplement: Supplementary file 1 — Supplementary Information [file 41467_2023_37744_MOESM1_ESM.pdf]
